# Huaier polysaccharides suppress triple-negative breast cancer metastasis and epithelial-mesenchymal transition by inducing autophagic degradation of Snail

**DOI:** 10.1186/s13578-021-00682-6

**Published:** 2021-09-04

**Authors:** Yuan Tian, Jin Wu, Lingjuan Zeng, Linxi Zhou, Ying Hu, Qinwen Pan, Wei Liu, Yuzhao Yan, Ziwei Wu, Zhaoyu Wang, Zhen Zeng, Peng Tang, Jun Jiang, Minghao Wang

**Affiliations:** 1 Breast Disease Center, Southwest Hospital, Army Medical University, 30# Gaotanyan street, Chongqing, 400038 China; 2 Department of General Surgery, Linyi People’s Hospital, Linyi, 276000 China

**Keywords:** Triple-negative breast cancer, Metastasis, Invasion, Huaier, In vitro, In vivo

## Abstract

**Background:**

Triple-negative breast cancer (TNBC) is the most aggressive subtype of breast cancer, and the targeted therapies are lacking for this type of cancer. We previously demonstrated that Huaier effectively improve 5-year OS and DFS in stage III TNBC patients, and the polysaccharides of Huaier (PS-T) have been identified as the major components of Huaier. However, the mechanisms of anti-tumor action of PS-T is unclear. This study aimed to investigate the effect of PS-T on TNBC cell invasion and migration.

**Results:**

This study showed that PS-T inhibited cell invasion and migration both in vitro and in vivo by inducing autophagy to suppress epithelial-mesenchymal transition (EMT). Autophagy inhibitor LY294002 or knockdown of ATG5 suppressed the inhibitory effects of PS-T. In addition, as a key transcription factor controlling EMT initiation, Snail was found to be degraded by PS-T induced autophagy. In addition, overexpression of Snail reversed the inhibitory effects of PS-T. Furthermore, it was confirmed that the expression of Snail was inversely correlated with LC3 and associated with poor prognosis using immunohistochemistry and TCGA database analysis, respectively.

**Conclusions:**

This study demonstrated that PS-T could inhibit EMT in breast cancer cells by inducing autophagy to degrade Snail protein, thus improving the prognosis of TNBC, offering potential treatment alternatives for TNBC patients.

**Supplementary Information:**

The online version contains supplementary material available at 10.1186/s13578-021-00682-6.

## Background

Triple-negative breast cancer (TNBC) is a subset of breast cancer that lacks the expression of estrogen receptor (ER) and progesterone receptor (PR), and has low level of human epidermal growth factor receptor 2 (HER2). It is usually associated with a higher risk of distant recurrence, higher rates of metastases, and worse overall survival (OS) compared to other subtypes [1, 2]. It is therefore critical to deepen our understanding of the TNBC metastatic mechanisms to find new therapeutic agents.

Increasing evidence showed that traditional Chinese medicines (TCMs) had a potential to inhibit tumor growth and therefore could be an alternative therapeutic avenue for cancer therapy [3, 4]. Specifically, Huaier exhibits a wide range of anti-cancer functions, including induction of apoptosis, inhibition of angiogenesis, and immune system stimulation, while no obvious side effects have been found [5, 6]. Our previous study showed that Huaier could effectively improve the 5-year overall survival (OS) and disease-free survival (DFS) in patients with stage III TNBC [7]. Due to this therapeutic significance, an increasing number of studies have focused on the potential molecular targets and mechanisms that underlie the anti-tumor effect of Huaier [5]. Polysaccharides of *Trametes robiniophila* *Murr* (PS-T) were identified as the major functional components of an aqueous Huaier extract [8], but the mechanism underlying the anti-cancer functions of PS-T in TNBC is still unclear.

Evidence indicates that the epithelial-mesenchymal transition (EMT) plays a pivotal role in cancer metastasis [9]. Recently, a few studies have investigated the role of autophagy in cell invasion and migration. Autophagy refers to a major cellular process in which a cell degrades long-lived proteins and cytoplasmic organelles [10]. Autophagy activation is an endogenous mechanism of suppressing tumor growth and metastasis through the degradation of Snail and Twist [11]. Nevertheless, the role of autophagy in EMT regulation and metastasis-related protein expression and function during tumorigenesis and cancer treatment is not clear.

In the present study, we aimed to investigate the mechanisms of anti-tumor effects of PS-T in TNBC. To this end, we have investigated the anti-metastasis effect of PS-T on TNBC both in vitro and in vivo. We have discovered a novel mechanism of the inhibition of tumor metastasis by PS-T; that is, PS-T may change EMT by inducing autophagy by degrading Snail protein. This is the first study to demonstrate that PS-T-induced autophagy suppresses cell invasion and migration through the degradation of Snail in TNBC cells, to the best of our knowledge. These results indicate that PS-T could be a valuable therapeutic adjuvant for the treatment of TNBC. Moreover, we suggest that targeting autophagy-dependent Snail degradation is a promising strategy for treatment of patients with TNBC.

## Results

### PS-T inhibits cell invasion, migration and EMT

**Fig. 1:**
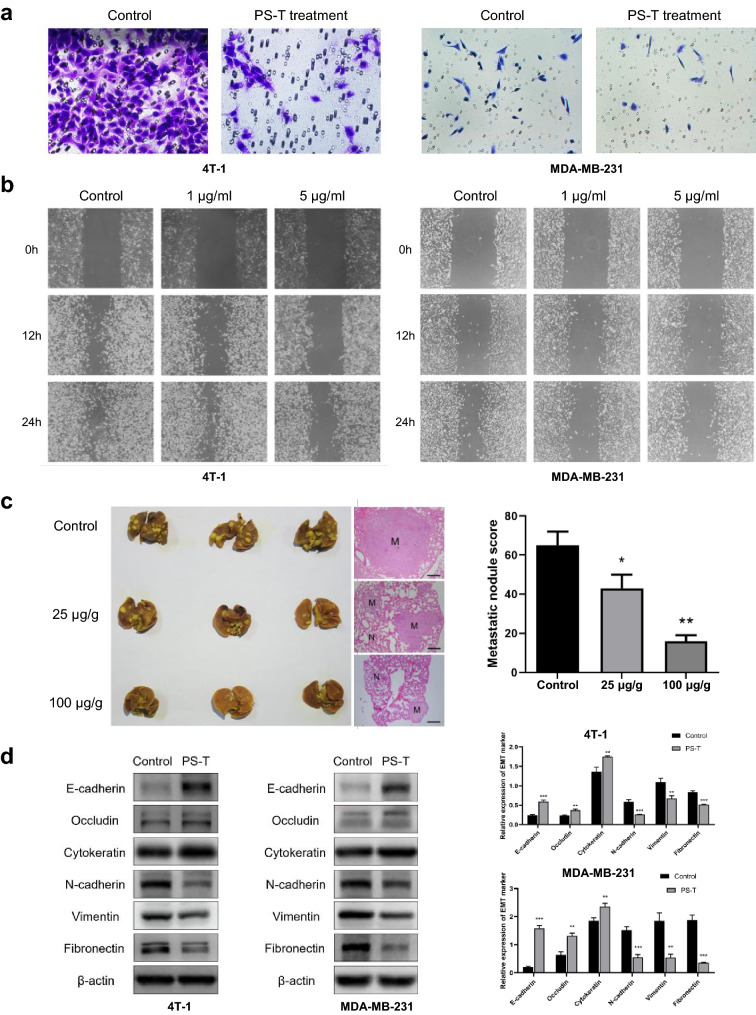
PS-T inhibits invasion, migration and EMT in breast cancer cells in vitro and in vivo. **a** The invasiveness of 4 T-1 and MDA-MB-231 cells after treatment with PS-T at 5 μg/mL for 12 h is evaluated using Transwell invasion assays at 24 h (mean  ±  SD, ***P  <  0.001). **b** Migratory ability of 4 T-1 and MDA-MB-231 cells after treatment with PS-T at different concentrations is evaluated using scratch assays at different time points (mean  ±  SD, **P  <  0.01, ***P  <  0.001). **c** Mice are treated with normal saline (control), 25 μg/g or 100 μg/g PS-T by oral gavage every other day for 21 days (mean  ±  SD, *P  <  0.05, **P  <  0.01, scale bars: 100 μm). **d** 4 T-1 and MDA-MB-231 cells are treated with 5 μg/mL PS-T for 24 h. The levels of each EMT marker are quantified using the NIH ImageJ software. (mean  ±  SD, *P  <  0.05 and **P  <  0.01)

We determined whether PS-T could suppress cell invasion and migration using transwell and scratch assays. 4 T-1 cells and MDA-MB-231 cells were incubated in media containing 5 μg/mL PS-T for 12 h before the transwell assays. PS-T produced no inhibitory effects on cell growth at these concentrations (Additional file 1: Figure S1a). Using the transwell assay, we found that PS-T treatment reduced cell motility. (Fig. 1a, Additional file 1: Figure S1b). Moreover, PS-T also inhibited cell migration in a dose-dependent manner in scratch assays (Fig. 1b, Additional file 1: Figure S1c). Furthermore, the anti-metastatic effect of PS-T on breast cancer in a mouse lung metastasis model was explored. The number and size of lung metastatic nodules was decreased in PS-T-treated mice in a dose-dependent manner, indicating the suppression of tumor metastasis (Fig. 1c). Based on these results, we concluded that PS-T could inhibit breast cancer metastasis in vitro and in vivo.

EMT plays an essential role in cancer invasion and metastasis. Therefore, we examined the impact of PS-T on the expression of proteins implicated in the process of EMT. As shown in Fig. 1d, upon treatment with PS-T, epithelial markers were upregulated and mesenchymal markers were downregulated. These data demonstrate that PS-T reversed EMT in breast cancer cells.

### PS-T reverses EMT by inducing autophagy

**Fig. 2:**
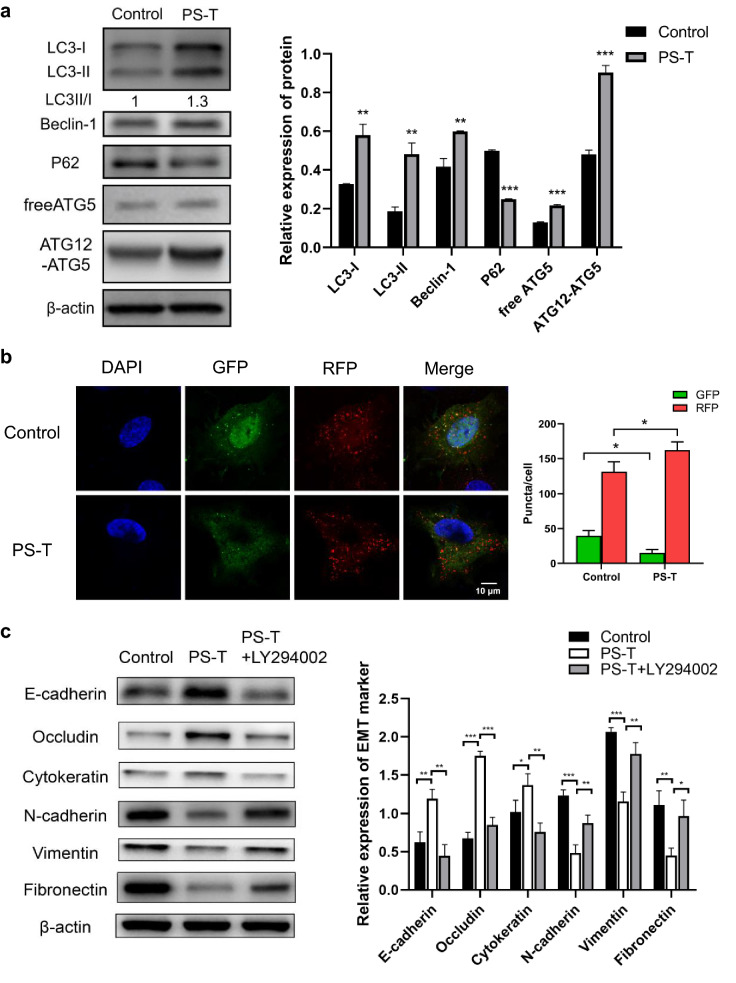
PS-T reverses EMT by inducing autophagy. **a** MDA-MB-231 cells are treated with 5 μg/mL PS-T for 24 h. The levels of each autophagy marker in MDA-MB-231 cells are quantified using the NIH ImageJ software. (mean  ±  SD, *P  <  0.05 and ***P  <  0.001). **b** Confocal microscopy images of cells treated with or without PS-T (5 μg/mL) for 24 h after transfection with mRFP-GFP-LC3 plasmid (scale bars: 10 μm). Quantification of LC3-GFP and LC3-RFP puncta/cells from three independent experiments (***P  <  0.001). **c** MDA-MB-231 cells are treated with PS-T (5 μg/mL) for 24 h with or without LY294002 (10 µM). The levels of each EMT marker in MDA-MB-231cells were quantified using NIH ImageJ software. (mean  ±  SD, *P  <  0.05 and **P  <  0.01)

According to previous studies, autophagy could contribute to the reversal of EMT and inhibit metastasis in cancer cells [12, 13]. It was found that autophagy induced by Huaier significantly suppressed the proliferation of cancer cells [14–16]. Using GEPIA 2.0 and bc-GenExMiner 4.5 to analyze TCGA databases, we found that the mRNA expression of the autophagy-related markers LC3B and Beclin-1 was positively correlated with E-cadherin expression in all breast cancer and TNBC patients (Additional file 2: Figure S2a, b). To investigate whether autophagy was involved in PS-T inhibition of EMT in breast cancer cells, the expression of the essential autophagy-associated proteins and autophagosome formation were detected. The upregulation of LC3-II/LC3-I, Beclin-1 and the downregulation of P62 in MDA-MB-231 cells after treatment with PS-T for 24 h, indicated an increase in autophagic activity (Fig. 2a). Notably, the upregulation of Beclin-1 suggests that PS-T upregulation of autophagy may be initiated by the upstream signaling. Because ATG12 conjugates with ATG5 to form an ATG12–ATG5 complex that is essential for autophagy, we also detected the expression of free ATG5 and ATG12‐ATG5 complex. As shown in Fig. 2a, PS-T treatment upregulated the free ATG5 (32 kDa) and ATG12–ATG5 complex (55 kDa).

To further validate the impact on autophagic flux, we took advance of the tandem mRFP-GFP-LC3 reporter assay [17]. PS-T treatment for 24 h resulted in an increase in the percentage of autolysosomes (GFP − /RFP  +  dots) (Fig. 2b). Hence, our results strongly suggest that PS-T promotes autophagy and enhances autophagosome–lysosome fusion in breast cancer cells.

To determine whether autophagy induced by PS-T contributes to EMT, we examined the EMT-related protein levels in MDA-MB-231 cells after PS-T treatment with or without LY294002. As shown in Fig. 2c, the epithelial markers were significantly increased and the mesenchymal markers decreased when autophagy was induced by PS-T. Conversely, the expression of EMT-related proteins was significantly reversed by the addition of the autophagy inhibitor LY294002. Taken together, these data indicate that PS-T reverses the EMT in breast cancer cells via induction of autophagy.

### ATG5 knockdown suppresses the inhibitory effects of PS-T

To verify the role of autophagy in PS-T inhibition of EMT transformation, we constructed a cell line, MDA-MB-231-siATG5, in which the autophagy key protein ATG5 was stably downregulated (Additional file 3: Figure S3). The expression levels of free ATG5 and ATG12–ATG5 complex in MDA-MB-231-siATG5 cells were significantly decreased, and PS-T-induced autophagy was suppressed after ATG5 knockdown (Additional file 4: Figure S4).

**Fig. 3:**
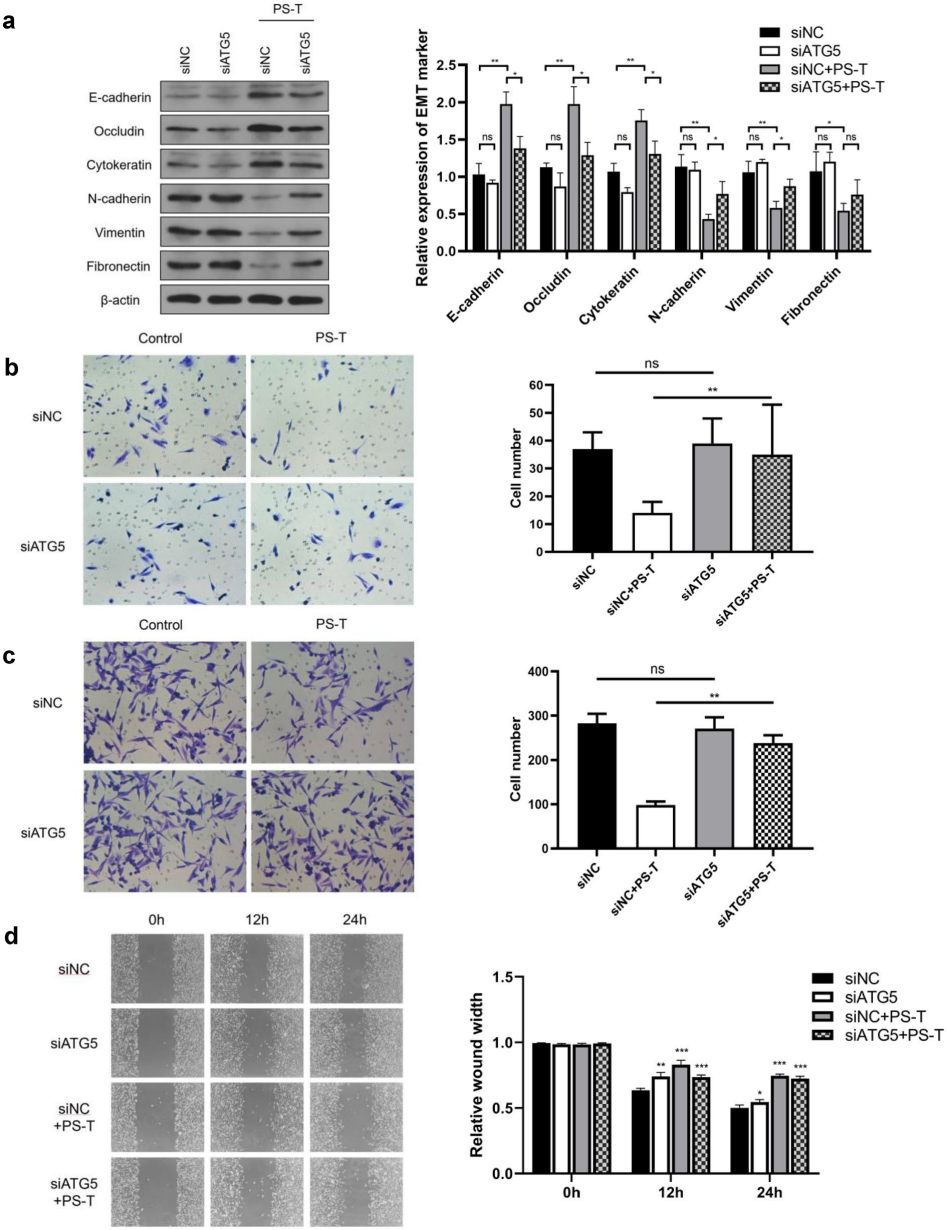
ATG5 knockdown suppresses the inhibitory effects of PS-T. **a** MDA-MB-231-siNC cells and MDA-MB-231-siATG5 cells are treated with or without PS-T (5 μg/mL) for 24 h. The levels of each EMT marker in MDA-MB-231cells are quantified using NIH ImageJ software. (mean  ±  SD, *P  <  0.05 and **P  <  0.01). **b** The invasiveness of MDA-MB-231-siNC cells and MDA-MB-231-siATG5 cells treated with PS-T at 5 μg/mL is evaluated using Transwell invasion assays at 24 h (mean  ±  SD, **P  <  0.01). **c**, **d** Migratory ability of MDA-MB-231-siNC and MDA-MB-231-siATG5 cells treated with PS-T at 5 μg/mL is evaluated using Transwell migration assays (**c**) and scratch assay (**d**) at 24 h (mean  ±  SD, *P  <  0.05, **P  <  0.01, and ***P  <  0.001)

We then observed changes in EMT-related protein expression after ATG5 knockdown. The results showed that PS-T can significantly inhibit EMT in MDA-MB-231-siNC cells. However, this effect was significantly reduced in the MDA-MB-231-siATG5 group (Fig. 3a). This indicates that PS-T-induced autophagy is involved in the inhibition of EMT transformation in MDA-MB-231 cells.

Furthermore, we verified that ATG5 knockdown reversed the inhibitory effect of PS-T on cell invasion and migration, as shown in Fig. 3b, c. The results were also confirmed using scratch assay, as shown in Fig. 3d. Taken these findings together, we concluded that PS-T-induced autophagy is involved in its inhibition of EMT transformation in MDA-MB-231 cells, which affects invasion and migration.

### PS-T induced autophagy reverses EMT by degrading Snail

**Fig. 4:**
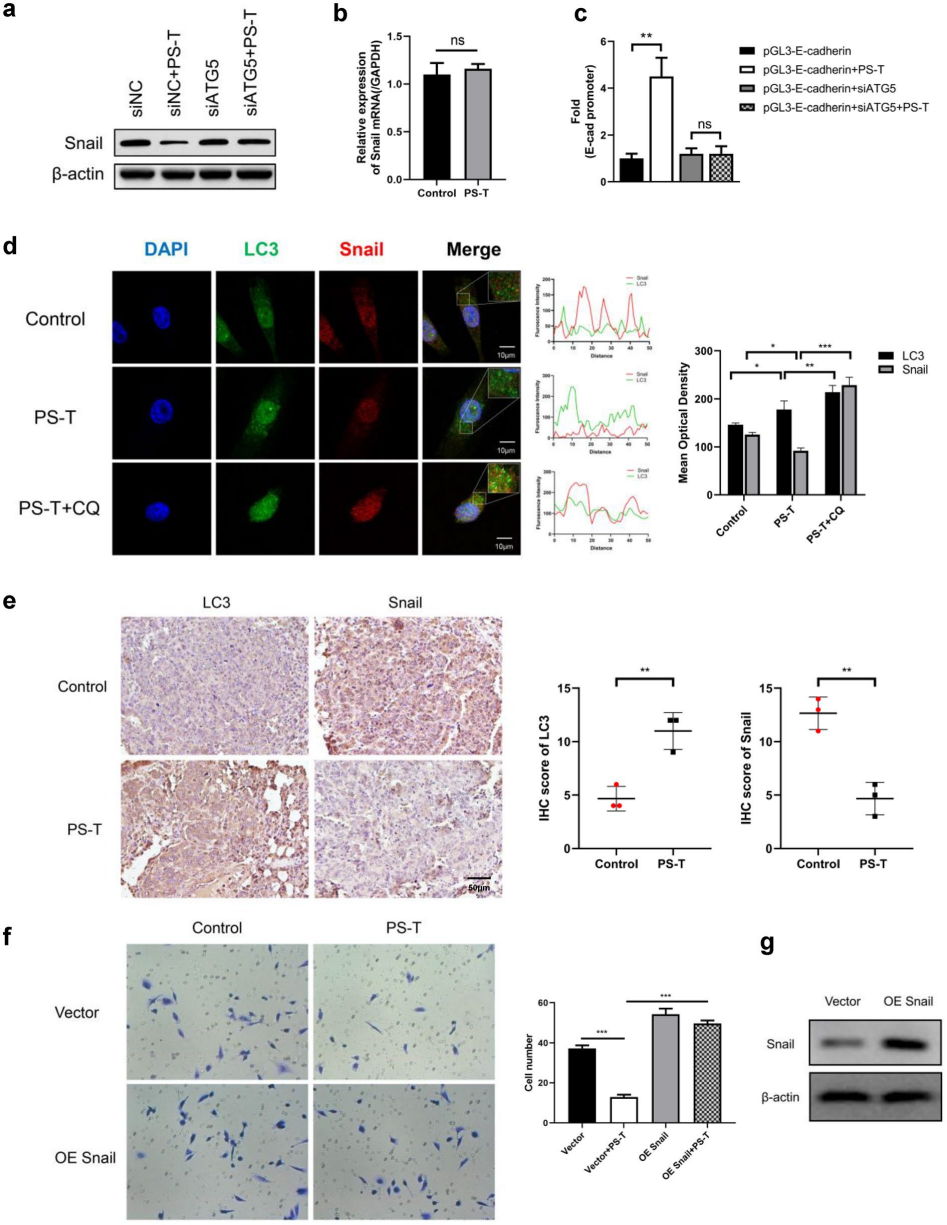
PS-T-induced autophagy reverses EMT by degrading Snail. **a** Protein levels of Snail in MDA-MB-231-siNC and MDA-MB-231-siATG5 cells treated with or without PS-T are evaluated using western blotting. **b** The mRNA levels of Snail in MDA-MB-231-siNC and MDA-MB-231-siATG5 cells treated with or without PS-T are evaluated using RT-qPCR. **c** The E-cadherin promoter reporter gene plasmid pGL3-E-cadherin is constructed and a double luciferase experiment is performed. The firefly/renilla fluorescence ratio is measured (mean  ±  SD, **P  <  0.01).** d** The colocalization and protein levels of LC3 and Snail in MDA-MB-231 cells treated with or without PS-T are tested using immunocytochemistry and assessed quantitatively. The mean optical density is measured and calculated to indicate protein levels. (mean  ±  SD, *P  <  0.05, **P  <  0.01, ***P  <  0.001, scale bars: 10 μm).** e** Expression of LC3 and Snail in the lung metastasis nodules of breast cancer mouse models are detected using immunohistochemistry (magnification  ×  20, scale bars: 50 μm). **f**, **g** MDA-MB-231 cell line overexpressing Snail is constructed by transfecting cells with pcDNA3.1-Snail. The Transwell invasion assay is performed to verify the invasiveness of cells (mean  ±  SD, ***P  <  0.001)

During EMT and cancer metastasis, expression of E-cadherin significantly decreases due to transcriptional regulation by Snail [18–24]. Thus, we explored whether Snail expression could be regulated by PS-T. Snail protein levels were decreased, but its mRNA levels did not change significantly when autophagy was induced by PS-T (Fig. 4a, b). Moreover, this autophagy-dependent Snail degradation was significantly decreased in MDA-MB-231-siATG5 cells. This result indicated that PS-T-induced autophagy can degrade Snail protein in breast cancer cells.

In order to confirm whether PS-T-induced autophagy affected the expression of E-cadherin at the transcriptional level, we constructed the E-cadherin promoter reporter gene plasmid pGL3-E-cadherin and performed a double luciferase experiment. The results showed that the firefly/renilla fluorescence ratio of the 5 μg/mL PS-T treatment group was significantly increased by nearly five-fold. However, this effect almost disappeared in the MDA-MB-231-siATG5 cells (Fig. 4c). It is suggested that PS-T-induced autophagy can regulate the transcription level of E-cadherin.

To further verify selective Snail degradation by PS-T induced autophagy, the expression of Snail and LC3 was evaluated using immunocytochemistry and assessed quantitatively (Fig. 4d). We found that LC3 was upregulated after PS-T treatment. Snail was downregulated and colocalized with LC3 by PS-T induced autophagy. However, in the MDA-MB-231-siATG5 cells, autophagy induced by PS-T failed to degrade Snail effectively (Additional file 5: Figure S5). These results further indicated that autophagy induced by PS-T degraded Snail specifically in an LC3 dependent manner.

Next, we detected the expression of LC3 and Snail protein in the lung metastasis nodules of the mouse models. As shown in Fig. 4e, LC3 protein was upregulated while Snail was downregulated in the 100 μg/g PS-T treatment group. These results demonstrated that PS-T inhibited metastasis by inducing autophagy and degrading Snail.

Furthermore, overexpression of Snail via transfection with pcDNA3.1-Snail attenuated the inhibitory effect of PS-T on MDA-MB-231 invasion, as shown in Fig. 4f, g. This result indicated that Snail plays a major role in PS-T-mediated tumor suppression. Taken together, these findings confirmed that PS-T regulates the invasion ability of breast cancer cells by degrading Snail protein.

### LC3 expression is inversely correlated with that of Snail and prognosis in TNBC

In order to investigate the potential clinical utility of Huaier in the treatment of TNBC, protein expression patterns of Snail were first analyzed by the Human Protein Atlas and immunohistochemistry (IHC). As was shown in Additional file 6: Figure S6a, b, we observed different expression levels of Snail protein in breast cancer tissues. In addition, mRNA expression of Snail was found to be significantly upregulated in TNBC compared to other subclasses measured by bc-GenExMiner 4.5. Furthermore, we used the Kaplan–Meier plotter to analyze the prognostic values of the mRNA expression of Snail in TNBC. As shown in Additional file 6: Figure S6d, higher Snail mRNA expression was associated with poorer prognosis in TNBC.

**Fig. 5:**
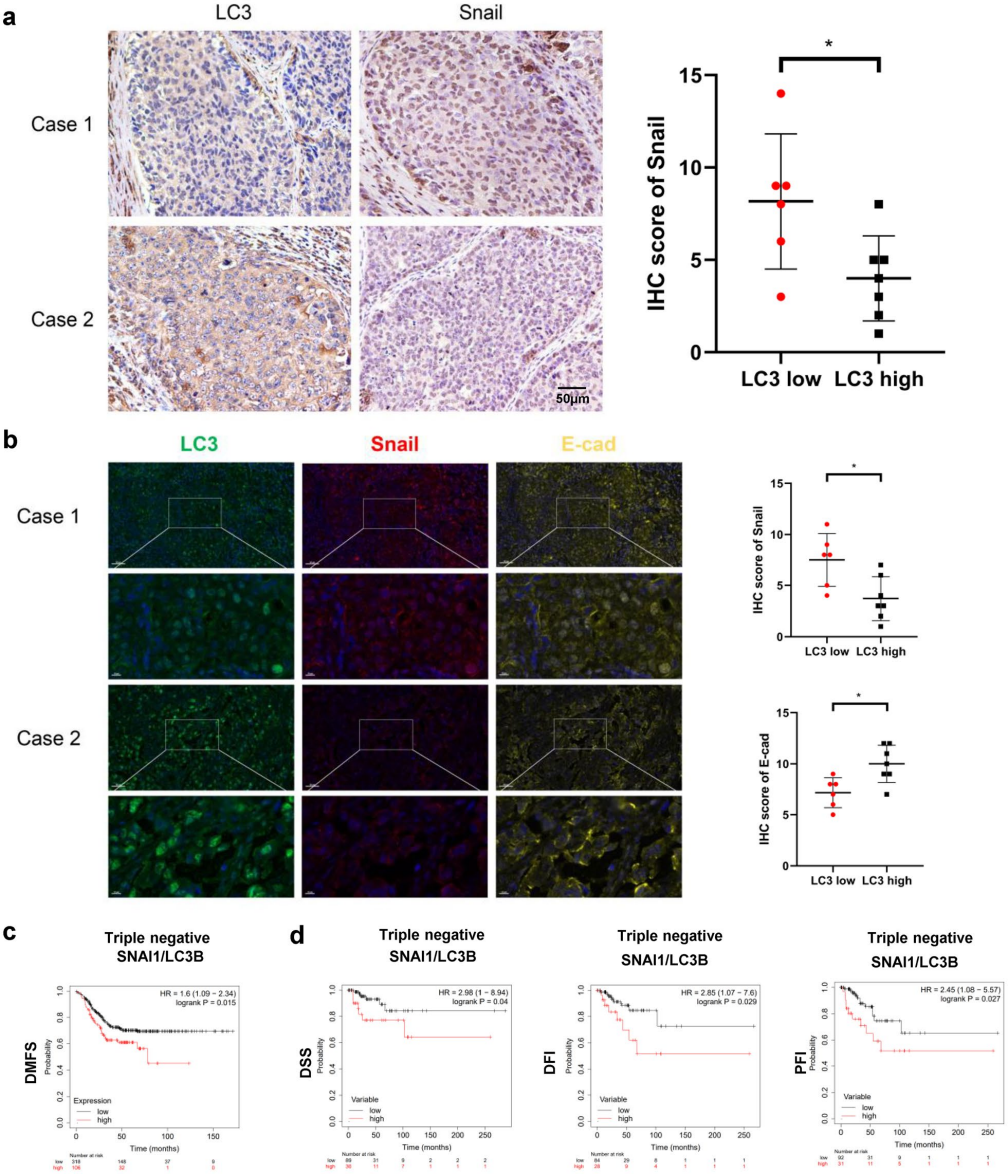
LC3 expression is inversely correlated with that of Snail and prognosis in TNBC. **a** Expression of LC3 and Snail in 13 TNBC tissue samples is detected using mmunohistochemistry (mean  ±  SD, magnification  ×  10, scale bars: 50 μm, *P  <  0.05). **b** Expression of LC3, Snail, and E-cadherin in 13 TNBC tissue samples is detected using multicolor immune fluorescence (mean  ±  SD, magnification  ×  20, scale bars: 50 μm, magnification  ×  69.1, scale bars: 10 μm, *P  <  0.05). **c**, **d** The prognostic values of the mRNA expression ratio of Snail/LC3 in TNBC patients are analyzed (Kaplan–Meier plotter). A higher Snail/LC3 mRNA expression ratio is associated with poorer distance metastasis free survival (P  =  0.015) in the Gene-Chip Datasets (**c**) and poorer disease specific survival (P  =  0.04), disease free interval (P  =  0.029) and progression free interval (P  =  0.027) in TCGA RNA-Seq datasets (**d**). The top quartile was used as the cutoff value

We further investigated the protein expression patterns of LC3 and Snail in 13 cases of TNBC using IHC. As shown in Fig. 5a, Snail was downregulated in the LC3 high group. This result was further verified using multicolor immunofluorescence analysis. We also observed upregulated expression of E-cadherin protein in the LC3 high group, while the expression of Snail was downregulated (Fig. 5b). In addition, a high ratio of Snail/LC3 expression was confirmed to be significantly associated with poor prognosis in TNBC (Fig. 5c, d). These results indicated that autophagy was inversely correlated with Snail expression and positively correlated with E-cadherin expression and prognosis in TNBC.

## Discussion

Treatment strategies for triple-negative breast cancer (TNBC) are limited, and the prognosis is extremely poor. Previous studies confirmed that about 35% of patients with TNBC developed distant metastases within 5 years of initial diagnosis, due to the ineffectiveness of current treatments as well as more malignant behaviors [25–27]. Studies have validated that EMT plays a critical role in cancer metastasis. EMT changes the expression of epithelial markers to a mesenchymal pattern and endows cells with enhanced migratory and invasive abilities [28]. Several studies have demonstrated that the anti-metastasis effect of Huaier was mediated, at least in part, by the suppression of EMT [29–34]. Our study showed that treatment with PS-T inhibited cell invasion and migration through the downregulation of EMT. In animal model experiments, lung metastatic nodules were reduced in mice treated with PS-T. These results were sufficient to demonstrate that PS-T can effectively reduce the metastatic ability of TNBC cells in vivo and in vitro. Further study of the specific molecular mechanism is necessary.

Interestingly, increasing evidence highlights the essential and multifaceted impact of autophagy in cancer. Autophagy seems to function as a tumor suppressor and inhibits tumorigenesis during tumor development [35–37]. Therefore, autophagy represents an attractive therapeutic target for cancer therapy. It was found that autophagy induced by Huaier significantly inhibited the proliferation of cancer cells [14–16]. Yang et al. confirmed that Huaier increased the expression of LC3 and induced the formation of autolysosomes in hepatoma SK-HEP-1 cells [16]. Similarly, our study showed that PS-T enhanced autophagosome–lysosome fusion and promoted autophagy in breast cancer cells (Fig. 2).

Previous studies found that autophagy inhibited EMT and metastasis in gastric cancer [12]. In addition, autophagy impaired the invasion migration and of glioblastoma cells by downregulating Snail and Slug [38]. Other studies suggested that autophagy reverses EMT and metastasis via the degradation of Snail and Twist [11]. Moreover, silencing autophagy with a chemical inhibitor or by gene silencing increased Snail levels and the mesenchymal phenotype was restored by blocking autophagy-related proteins [13, 38]. These studies all indicate that autophagy might play a crucial role in regulating Snail, which is essential for determining EMT in cancer. In our research, we identified for the first time that PS-T-induced autophagy inhibits cancer invasion and migration by the degradation of Snail protein (Fig. 3, Fig. 4).

Snail/SNAI1 is a key transcription factor in the EMT process [18–20]. It represses the E-cadherin/CDH1 expression and subsequently promotes cancer metastasis [21–24]. Our study showed that overexpression of Snail inhibited the transcription of E‐cadherin, induced EMT, and favors tumor metastasis (Fig. 4). In addition, it has been shown that increased levels of Snail inhibit the activity of p53, and also induce acquisition of stem cell characteristics [39, 40]. These results indicate that the inactivation of Snail proteins could be a promising therapeutic target for cancer.

To investigate the potential application of PS-T in the clinical treatment of TNBC, we explored the relationship between Snail expression and prognosis (Fig. 5). We found that mRNA expression of Snail was significantly higher in TNBC than in other subclasses of breast cancer. Simultaneously, high Snail staining in TNBC tissues was significantly inversely correlated with that of LC3. Furthermore, we found that elevated expression of Snail or the ratio of Snail/LC3 was significantly associated with poorer prognosis of TNBC. These lines of evidence suggest that autophagy degrades Snail in suppressing metastasis and supports the therapeutic value of PS-T in TNBC treatment.

## Conclusions

**Fig. 6:**
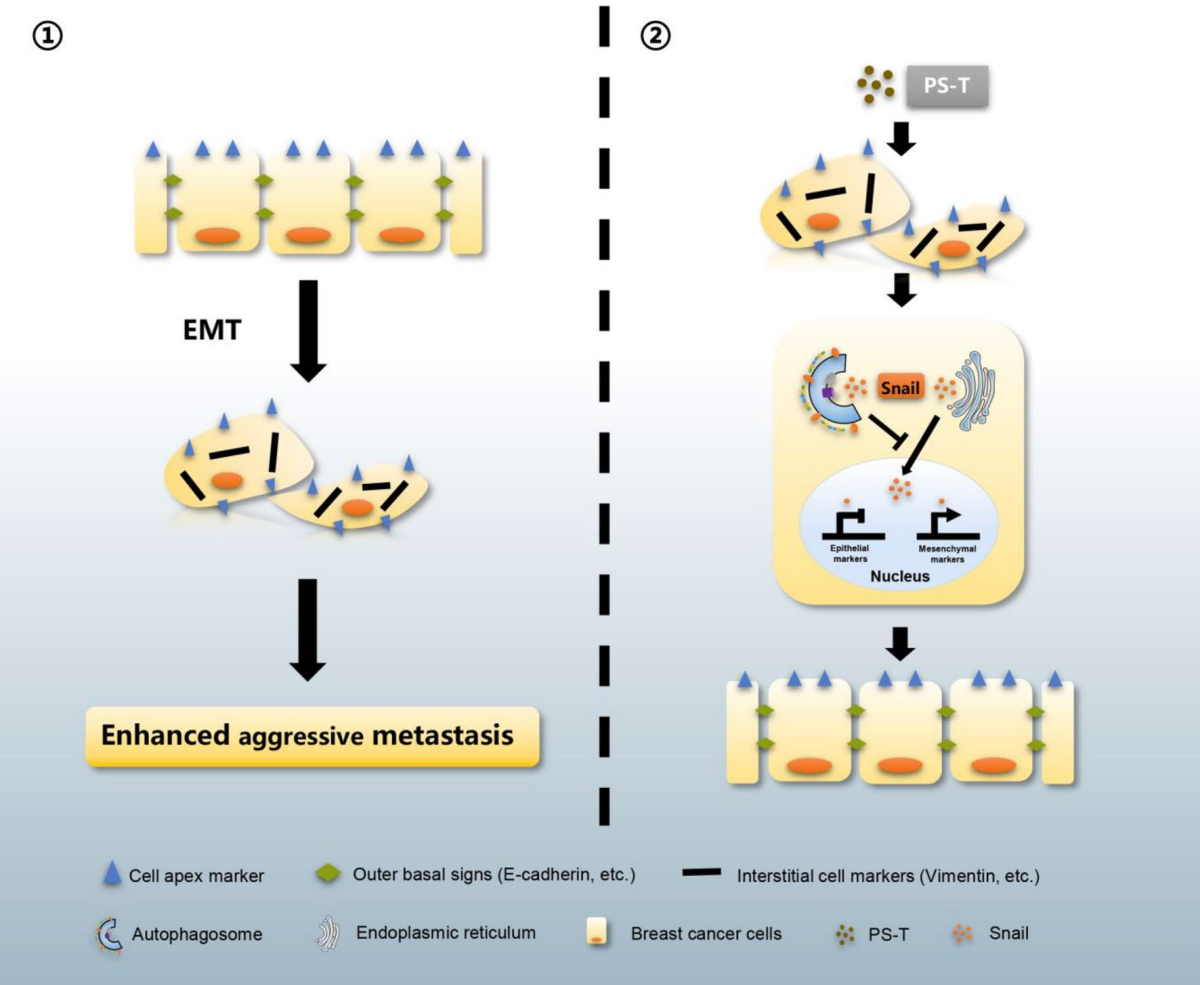
Proposed model illustrating the effect of PS-T induced autophagy on Snail degradation to reverse epithelial-mesenchymal transition. When autophagy is induced by PS-T, degradation of Snail protein by autophagy represses epithelial-mesenchymal transition to contribute to the acquisition of an epithelial-like phenotype

Taken together, our results demonstrate that PS-T can suppress EMT in breast cancer cells by inducing autophagy to degrade Snail protein, which aids in both EMT and metastasis (Fig. 6). This study provides new insights into the mechanism by which PS-T reduces the recurrence of TNBC and new ideas for comprehensive treatment strategies for TNBC patients.

## Materials and methods

### Chemicals and reagents

Huaier crude extract was obtained from Qidong Gaitianli Pharmaceutical Co., Ltd. (Qidong, China). Polysaccharides of *Trametes* *robiniophila* *Murr* (PS-T) were isolated and purified as previously reported by our team [41]. The purity of the PS-T was over 99%, which was evaluated using a phenol–sulfuric acid method with glucose as the standard [42]. Primary antibodies against MAP1LC3B (NB100-2220), Snail (NBP2-50300) were purchased from Novus Biologicals. E-cadherin (Ab40772), Cytokeratin (Ab7753), Vimentin (Ab8978) and ATG12 Antibody (ab155589) were from Abcam. ATG5 Antibody (PA5-23186) were from Thermo Fisher. N-cadherin (AF4039), Fibronectin (AF5335), Occluding (AF7504), P62 (AF5128), Beclin-1 (AF5128) and β-actin (AF7018) was purchased from Affinity. SNAI1 (SC-271977), and E-cadherin (SC-7870) were from Santa Cruz Biotechnology. Secondary antibodies against rabbit IgG (7074) were from Cell Signaling Technology. Secondary antibodies for immunocytochemistry (FITC and Cy3) were purchased from EarthOx. Other materials were obtained from commercially available chemicals.

### Cell culture and CCK-8 assay

Human breast cancer MDA-MB-231 cells, mouse breast cancer 4 T-1 cells and human embryonic kidney HEK-293 T cells were purchased from FuHeng Cell Center, Shanghai, China. The cells were cultured according to their instructions. Cell viability was investigated by the CCK-8 assay. The experiment was conducted in accordance with the instructions.

### Scratch assay, transwell migration and matrigel invasion assays

Confluent monolayers of cells were scratched with a 200 μL pipette tip. Cells were cultured in serum-free medium with or without PS-T (1 μg/mL or 5 μg/mL). The width of the scratch was measured using an Olympus inverted microscope at 0 h, 12 h, and 24 h. For the migration assay, transwell filters purchased from Corning (Corning Costar, USA) were used. Cells were incubated in serum-free medium with or without PS-T (5 μg/mL) for 12 h, then we suspended the cells (5  ×  10^4^) in 400 μL of serum-free medium and added to the upper chamber. Then, complete medium containing 10% fetal bovine serum was added to the lower chamber. After incubated at 37 °C for 24 h, the cells were stained with crystal violet and counted in three random fields. For the invasion assay, the transwell filter were covered with Matrigel (Corning, 354480).

### In vivo* metastasis assays*

Six-week-old Balb/c female mice were purchased from the Model Animal Research Center of Nanjing University. All mice were housed for 3 days to acclimatize them to the conditions. 4 T-1 cells were injected into the lateral tail vein (1 × 10^6^ cells per mouse). Mice were randomly divided into three groups (control, 25 μg/g PS-T, 100 μg/g PS-T) with five mice per group. The next day, the drug was administered by oral gavage every other day. The control group was treated with 100 μL of normal saline. Mice were sacrificed after 21 days. Lungs were harvested and fixed with Bouin’s solution. The lung metastatic nodules were counted by the naked eye. The scale includes four grades: grade I (nodule diameter  <  0.5 mm), grade II (0.5 mm  ≤  nodule diameter  <  1 mm), grade III (1 mm  ≤  nodule diameter  <  2 mm), grade IV (nodules diameter  ≥  2 mm). Metastatic nodule score was calculated as follows: I  ×  1  +  II  ×  2  +  III  ×  3  +  IV  ×  4. All of the research involving animals complied with protocols approved by the Laboratory Animal Welfare and Ethics Committee of the Army Medical University (AMUWEC2019199).

### Western blotting

Cells were washed with cold PBS and then harvested. The protein was extracted and the concentrations were determined using the Pierce BCA Protein Assay Kit (Thermo Scientific), then stored at  − 20 °C. Proteins were electrophoresed by SDS-PAGE and transferred to PVDF membranes. After blocking with 5% non-fat milk for 2 h at room temperature, the PVDF membranes were incubated with primary antibodies overnight at 4 °C and secondary antibodies for 1 h at room temperature. Signals were detected by an ECL detection system, and evaluated using ImageJ software.

### mRFP-GFP-LC3 fluorescence for tracing autophagic flux

MDA-MB-231 cells were transfected with mRFP-GFP-LC3 plasmid according to the manufacturer's instructions and treated with 5 μg/mL PS-T or PBS for 24 h. Autophagy. The RFP and GFP puncta in cells was observed using laser confocal microscopy and counted to evaluate the level of autophagy.

### Construction of ATG5 stable interference cell line

Three siRNAs targeting ATG5 mRNA at positions 393, 443, and 853 were designed. The interference efficiency of targeting small fragments was analyzed by qPCR. The results showed that siRNA targeting 853 was the most efficient, reaching 70% of interference. A small fragment of siRNA was inserted into the pLVX-ShRNA2-Puro plasmid to construct a lentiviral recombinant plasmid. The constructed plasmid was named pLVX-ShRNA2 -Puro-Homo-ATG5-853, and it was packaged and transferred into HEK293 cells. Transfection efficiency was observed at 24 and 72 h after transfection, and the lentivirus pLVX-ATG5 titer was measured as 8  ×  10^7^ TU/mL. MDA-MB-231 cells were infected, and infection efficiency was observed after 48 h. We then screened positive cells using the optimal concentration of 0.2 μg/mL puromycin. Monoclonal cells were obtained by limiting dilution (Additional file 2: Figure S2).

### E-cadherin promoter activity assay

First, we looked up the information of E-cadherin on the chromosome in the Ensemble database (https://www.ensembl.org/index.html) and obtained the E-cadherin promoter sequence. pUC57-E-cadherin and pGL3-basic were double digested with SacI and HindIII, and then the recovered and purified target fragment E-cadherin (SacI/HindIII) was ligated to the vector pGL3-basic (SacI/HindIII), and the linked product was named pGL3 -basic-E-cadherin(Additional file 4: Figure S4). Then DH5a competent cells were transformed with the ligation product, coated with LB AMP  +  plates, and cultured in a 37 °C incubator overnight. A single colony was picked, inoculated in LB AMP  +  liquid medium, and cultured at 37 °C and 250 rpm overnight. The cultured bacteria liquid was used for colony PCR identification. Extraction was then performed using an endotoxin-free plasmid extraction kit and the extracts were stored at − 20 °C. MDA-MB-231 or MDA-MB-231-siATG5 cells were plated into 96-well plates at a volume of 3 × 10^4^ cells/well in a volume of 100 µL and incubated overnight at 37 °C in an incubator. Subsequently, Lipo3000 was used for plasmid transfection. After 6 h, the solution was changed and 5 µg/mL PS-T was added for stimulation. After 24 h, the intracellular dual fluorescence was detected using the Promega Dual-luciferase assay kit (E1910) kit. The relative expression of firefly and renilla luciferase was calculated.

### RNA isolation and quantitative RT-PCR

Total mRNA was isolated using Trizol (Invitrogen, Carlsbad, CA, USA), and followed by cDNA synthesis with Taq-Man Reverse an RNeasy kit (QIAGEN, Hilden, Germany). mRNA expression was detected using SYBR Premix Ex Taq II Kit (Takara, Kusatsu, Shiga Prefecture, Japan) in an Eppendorf Mastercycler realplex. The 2^−∆∆Ct^ formula was used to calculate the relative abundance of RNA genes compared with *GAPDH*.

### Immunofluorescence staining

Cells were cultured on coverslips for 24 h with or without 5 μg/mL PS-T. Cells were fixed with 4% (w/v) paraformaldehyde for 20 min and permeabilized with 0.5% Triton X-100 for 20 min at room temperature. Cells were incubated with primary antibodies overnight at 4 °C, followed FITC- or Cy3-conjugated secondary antibodies (1:100 in PBS) at room temperature for 60 min. The nuclear were counterstained using DAPI. For tissue immunofluorescence staining, rehydrated paraffin-embedded sections were microwaved in 10 mM sodium citrate buffer (pH 6) to unmask the antigen, and incubated with the primary antibody followed by Opal 520, 620, 570 secondary antibody for 60 min at room temperature. Images were captured by confocal microscopy (FV-1000; Olympus, Tokyo). The IPP6.0 software was used to perform optical density analysis on immunofluorescence photos. Three areas in 400 photos were selected per section to calculate mean optical density.

### Lentivirus transfection

First, we queried the NCBI (https://www.ncbi.nlm.nih.gov/) and found the human Snail protein. The information is as follows: GenBank: AF125377.1, 264AA, coding sequence is 795 bp. This fragment was cloned into the pcDNA3.1 plasmid by BamHI/HindIII double restriction digestion. After transformation, clone selection, identification and sequencing, it was determined that the construction was successful. Extraction was then performed using an endotoxin-free plasmid extraction kit. Store at − 20 °C. Lipo3000 was used for pcDNA3.1-Snail recombinant plasmid transfection.

### Bioinformatics

In this study, The Human Protein Atlas (https://www.proteinatlas.org) is used to compare protein expression of Snail in breast cancer tissues. The correlation between specific gene expression and survival curves in breast cancer was assessed by the GEPIA (http://gepia.cancer-pku.cn/), bc-GenExMiner 4.5 (http://bcgenex.ico.unicancer.fr), KM plotter (http://kmplot.com/analysis/). BRCA of TCGA was download from UCSC database (http://xenabrowser.net). We used the ratio of SNAI1/LC3B expression to stratify patients into high and low groups and looked for association with outcomes. In GEO gene-chip datasets, we used SNAI1(219480_at) as a numerator and LC3B(208786_s_at) as the denominator to calculate the SNAI1/LC3B ratio of each patient. In TCGA RNA-Seq datasets, we used the gene-level transcription estimates, as in log2(x  +  1) transformed RSEM normalized count to calculate the SNAI1/LC3B ratio of each patient.

### Immunohistochemistry (IHC) analysis

All patient samples were collected from the Breast Disease Center, First Affiliated Hospital of the Army Medical University (Chongqing, China). Specimens were fixed in 10% formalin and embedded in paraffin. After soaking in xylene to dewax and rehydrating using an ethanol gradient. The 4 μm slices were de-waxed, rehydrated and stained according to the SP kit instructions (ZSGB-BIO ORIGENE, SP-9000). IHC staining was scored according to the staining intensity score × the percentage of stained cells. The intensity of staining was scored according to 0 points: no staining, 1 point: weak positive, 2 points: moderate positive, 3 points: strong positive. The percentage of stained cells was counted 1 follows: 1–25%, 2 points: 26–50%, 3 points: 51–75% and 4 points: 76–100%.

### Statistical analysis

Statistical analyses were performed using GraphPad Prism 8. All data were presented as means  ±  standard deviation and analyzed using variance (ANOVA) or Student’s *t *test. All experiments were performed at least three times. P  <  0.05 was considered statistically significant.

## Supplementary Information

**Additional file 1: Figure S1.** PS-T reduces cell viability and inhibits invasion and migration in breast cancer cells. **a** Cytotoxicity of different concentrations of PS-T in 4T-1 and MDA-MB-231 cells detected using CCK-8 kit at 24 h. The viability of untreated cells is considered 100%. The experiments are performed in triplicates and data are presented as the mean ± SD of three separate experiments. **b** The invasiveness of 4T-1 and MDA-MB-231 cells after treatment with PS-T at 5 μg/mL is evaluated using Transwell invasion assays at 24 h (mean ± SD, *** P < 0.001). **c** The migratory ability of 4T-1 and MDA-MB-231 cells after treatment with PS-T at different concentrations is evaluated using scratch assays at different time points (mean ± SD, * P < 0.05, ** P < 0.01).

**Additional file 2: Figure S2.** mRNA expression of LC3B and Beclin-1 is positively correlated with that of E-cadherin in breast cancer. Using GEPIA 2.0 and bc-GenExMiner 4.5 to analyze TCGA databases, we found that the mRNA expression of the autophagy-related markers LC3B and Beclin-1 was positively correlated with E-cadherin expression in all breast cancer (**a**) and TNBC patients (**b**).

**Additional file 3: Figure S3.** Construction of a key autophagy protein ATG5 to stably interfere with ATG5 expression in cell lines MDA-MB-231-siATG5. **a** Three siRNAs targeting ATG5 mRNA 393, 443, and 853 sites are designed, and the interference efficiency of targeting small fragments is analyzed using qPCR. The siRNA targeting 853 sites has the highest efficiency, reaching 70% of interference. **b** Small siRNA fragment Homo-ATG5-853 is inserted into the pLVX-ShRNA2-Puro plasmid to construct a lentiviral recombinant plasmid. **c** pLVX-ShRNA2 -Puro-Homo-ATG5-853-1 plasmid digestion identification. The plasmid vector pLVX-ShRNA2-Puro originally had only one XhoI restriction site. When designing the interference fragment, the XhoI restriction site was artificially introduced. When the plasmid was able to produce 1400 bp DNA band when digested with XhoI, the target gene fragment was inserted into the plasmid vector pLVX-ShRNA2-Puro, which was named pLVX-ShRNA2-Puro-Homo-ATG5-853. **d** Plasmid sequencing report showing successful insertion of siRNA fragments. **e** Lentiviral packaging of the pLVX-ShRNA2-Puro-Homo-ATG5-853 recombinant plasmid is performed and transfected into HEK293 cells. Transfection efficiency is observed at 24 and 72 h after transfection (scale bars: 100 μm). **f** MDA-MB-231 cells are infected, and infection efficiency is observed 48 h later. The optimal concentration of 0.2 μg/mL puromycin is used to screen positive cells (scale bars: 100 μm).

**Additional file 4: Figure S4.** Verification of ATG5 knockdown effect in cell line MDA-MB-231-siATG5. **a** The expression levels of ATG5 in MDA-MB-231cells are analyzed using western blotting (mean ± SD, **P < 0.01). **b** The expression levels of autophagy-related markers in MDA-MB-231cells are analyzed using western blotting (mean ± SD, *P < 0.05, **P < 0.01, *** P < 0.001). **c** To further verify the effect of ATG5 knockdown on autophagy, the expression LC3 is evaluated using immunocytochemistry and assessed quantitatively (mean ± SD, *P < 0.05, scale bars: 10 μm).

**Additional file 5: Figure S5.** ATG5 knockdown suppresses Snail degradation by PS-T. **a** Protein levels of LC3 and Snail in MDA-MB-231-siNC cells and MDA-MB-231-siATG5 cells treated with or without PS-T analyzed using immunocytochemistry and assessed quantitatively. The mean optical density is measured to indicate protein levels. (mean ± SD, * P < 0.05 , scale bars: 10 μm).

**Additional file 6: Figure S6.** Snail is highly expressed in TNBC and correlates with cancer relapse. **a** Representative immunohistochemistry images show Snail protein expression in breast cancer tissues (Human Protein Atlas, scale bars: 50 μm). **b** Expression of Snail in 13 TNBC tissue samples is detected using immunocytochemistry. Representative images of Snail staining in breast cancer tissue; magnification ×10; scale bars: 100 μm. **c** Relationship between Snail mRNA expression and different subclasses of breast cancer patients (bc-GenExMiner 4.5) (* P < 0.05, ** P < 0.01, and *** P < 0.001). **d** The prognostic values of the mRNA expression of Snail in TNBC patients are analyzed (Kaplan-Meier plotter). The top quartile was used as the cutoff value.

## Data Availability

Additional figure and associated figure legends are provided in the supplemental material and are available online with the paper.
