# Supplementary figures and images for: Huaier polysaccharides suppress triple-negative breast cancer metastasis and epithelial-mesenchymal transition by inducing autophagic degradation of Snail
Source: Cell Biosci. 2021 Sep 4;11:170. doi: 10.1186/s13578-021-00682-6 (PMC8417980; doi:10.1186/s13578-021-00682-6)

**Fig. S1: PS-T reduces cell viability and inhibits invasion and migration in breast cancer cells.**

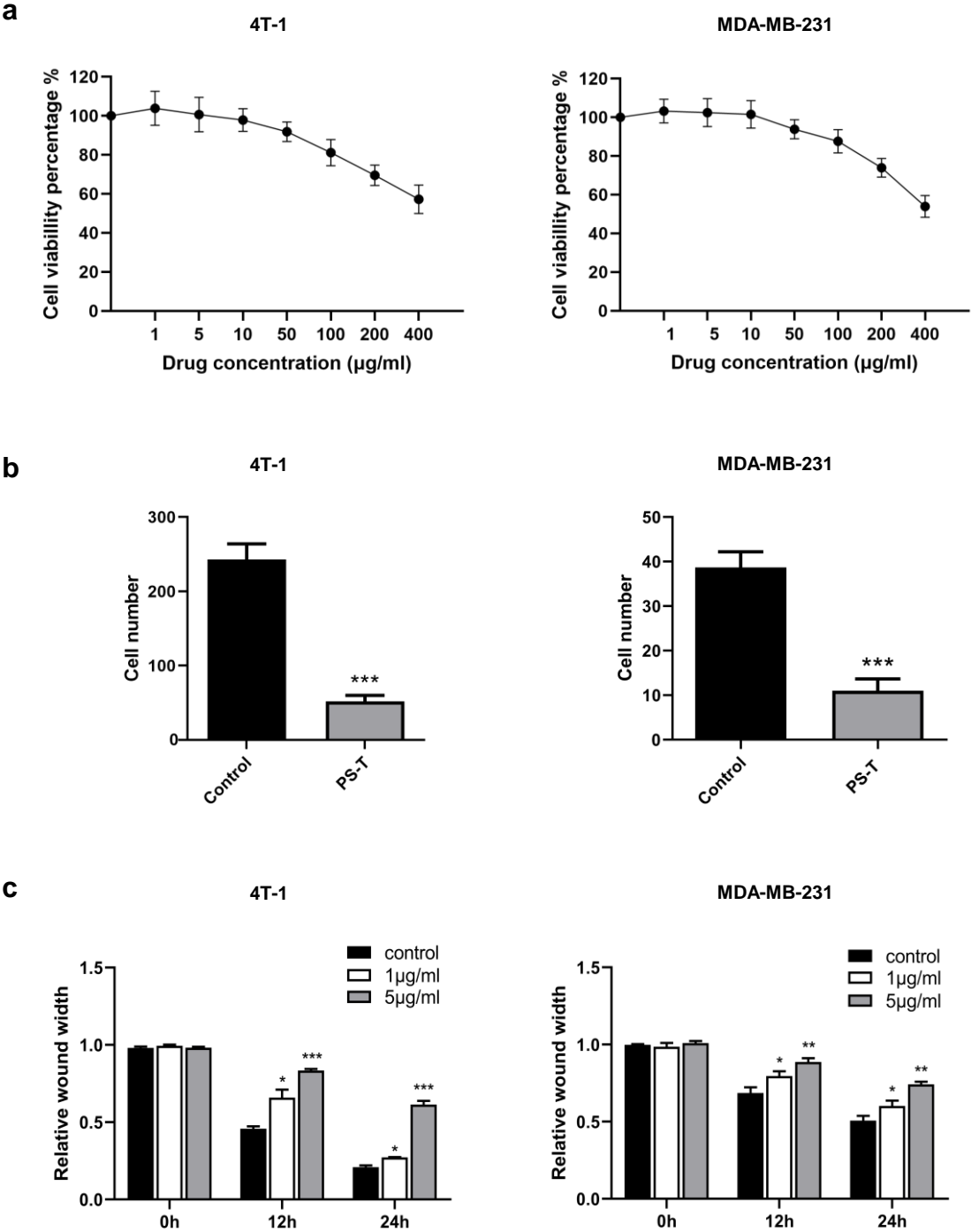

Supplement: Supplementary file 1 — Additional file 1: Figure S1. PS-T reduces cell viability and inhibits invasion and migration in breast cancer cells. a Cytotoxicity of different concentrations of PS-T in 4T-1 and MDA-MB-231 cells detected using CCK-8 kit at 24 h. The viability of untreated cells is considered 100%. The experiments are performed in triplicates and data are presented as the mean ± SD of three separate experiments. b The invasiveness of 4T-1 and MDA-MB-231 cells after treatment with PS-T at 5 μg/mL is evaluated using Transwell invasion assays at 24 h (mean ± SD, *** P < 0.001). c The migratory ability of 4T-1 and MDA-MB-231 cells after treatment with PS-T at different concentrations is evaluated using scratch assays at different time points (mean ± SD, * P < 0.05, ** P < 0.01). [file 13578_2021_682_MOESM1_ESM.pdf]

**Fig. S3: Construction of a key autophagy protein ATG5 knockdown stable cell line MDA-MB-231-siATG5.**

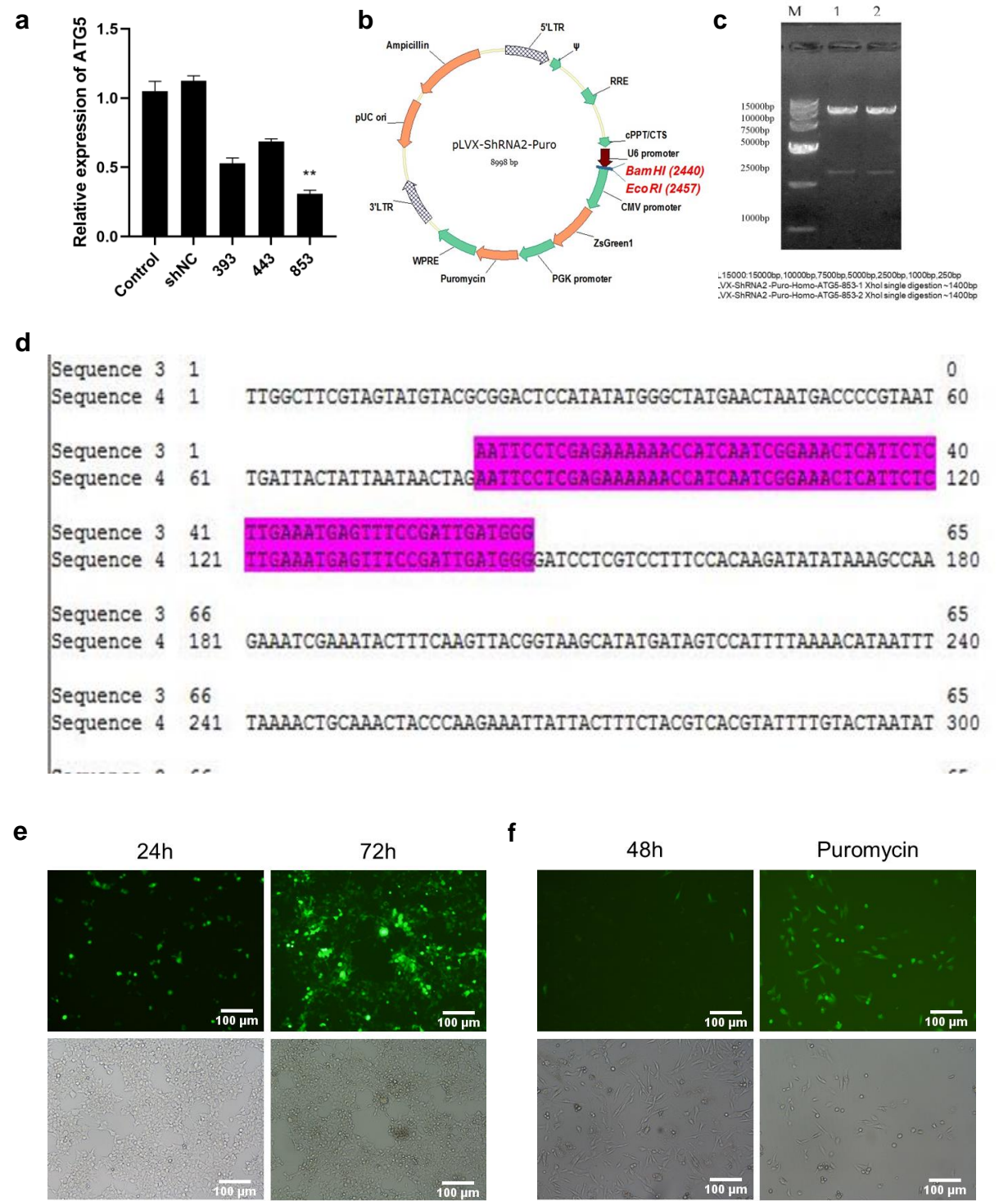

Supplement: Supplementary file 3 — Additional file 3: Figure S3. Construction of a key autophagy protein ATG5 to stably interfere with ATG5 expression in cell lines MDA-MB-231-siATG5. a Three siRNAs targeting ATG5 mRNA 393, 443, and 853 sites are designed, and the interference efficiency of targeting small fragments is analyzed using qPCR. The siRNA targeting 853 sites has the highest efficiency, reaching 70% of interference. b Small siRNA fragment Homo-ATG5-853 is inserted into the pLVX-ShRNA2-Puro plasmid to construct a lentiviral recombinant plasmid. c pLVX-ShRNA2 -Puro-Homo-ATG5-853-1 plasmid digestion identification. The plasmid vector pLVX-ShRNA2-Puro originally had only one XhoI restriction site. When designing the interference fragment, the XhoI restriction site was artificially introduced. When the plasmid was able to produce 1400 bp DNA band when digested with XhoI, the target gene fragment was inserted into the plasmid vector pLVX-ShRNA2-Puro, which was named pLVX-ShRNA2-Puro-Homo-ATG5-853. d Plasmid sequencing report showing successful insertion of siRNA fragments. e Lentiviral packaging of the pLVX-ShRNA2-Puro-Homo-ATG5-853 recombinant plasmid is performed and transfected into HEK293 cells. Transfection efficiency is observed at 24 and 72 h after transfection (scale bars: 100 μm). f MDA-MB-231 cells are infected, and infection efficiency is observed 48 h later. The optimal concentration of 0.2 μg/mL puromycin is used to screen positive cells (scale bars: 100 μm). [file 13578_2021_682_MOESM3_ESM.pdf]

**Fig. S4: Verification of ATG5 knockdown effect in cell line MDA-MB-231-siATG5**

**a**

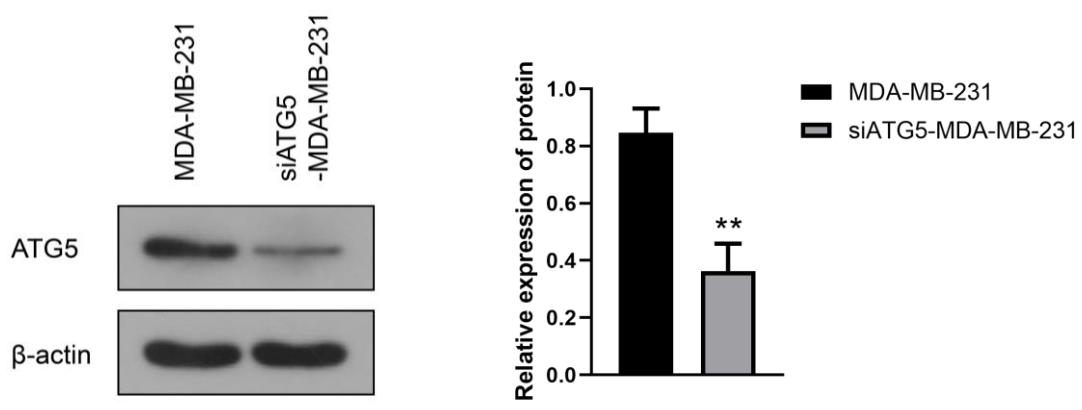

**b**

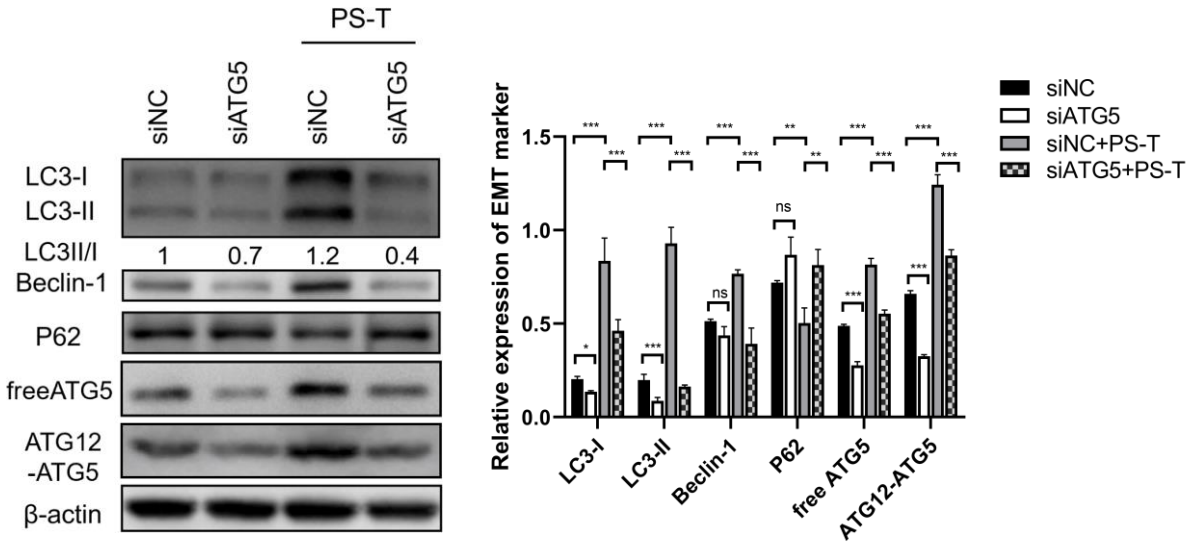

**c**

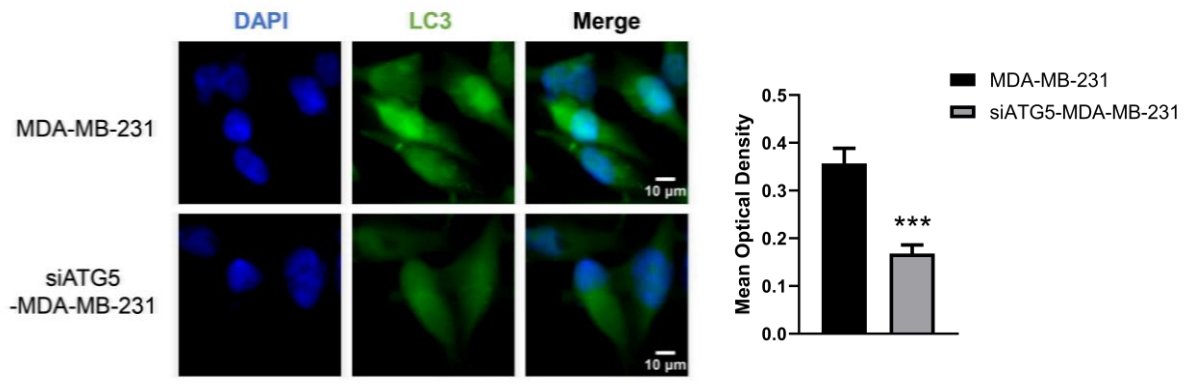

Supplement: Supplementary file 4 — Additional file 4: Figure S4. Verification of ATG5 knockdown effect in cell line MDA-MB-231-siATG5. a The expression levels of ATG5 in MDA-MB-231cells are analyzed using western blotting (mean ± SD, **P < 0.01). b The expression levels of autophagy-related markers in MDA-MB-231cells are analyzed using western blotting (mean ± SD, *P < 0.05, **P < 0.01, *** P < 0.001). c To further verify the effect of ATG5 knockdown on autophagy, the expression LC3 is evaluated using immunocytochemistry and assessed quantitatively (mean ± SD, *P < 0.05, scale bars: 10 μm). [file 13578_2021_682_MOESM4_ESM.pdf]

**Fig. S5: ATG5 knockdown suppresses Snail degradation by PS-T.**

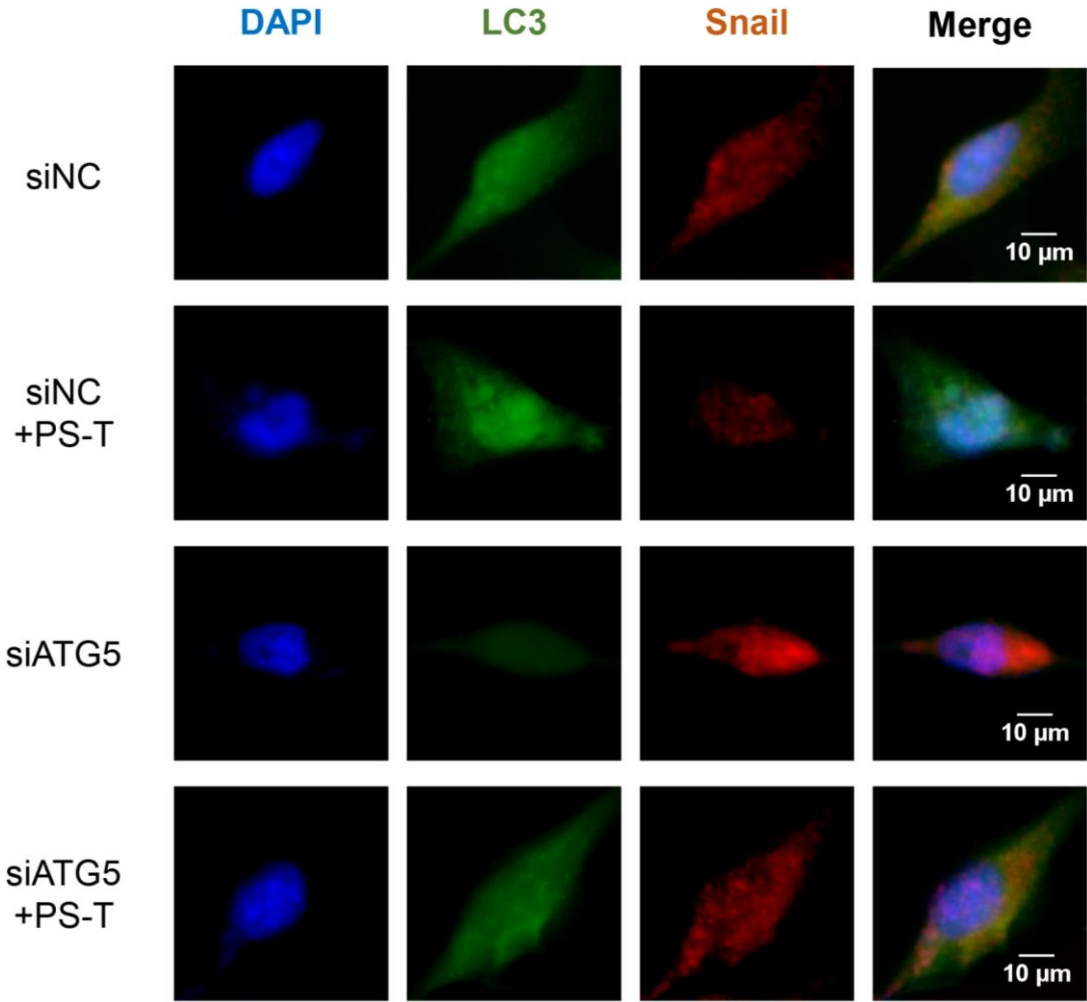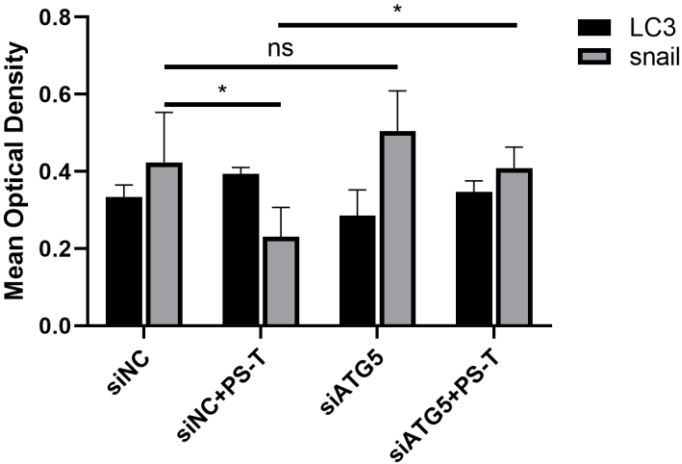

Supplement: Supplementary file 5 — Additional file 5: Figure S5. ATG5 knockdown suppresses Snail degradation by PS-T. a Protein levels of LC3 and Snail in MDA-MB-231-siNC cells and MDA-MB-231-siATG5 cells treated with or without PS-T analyzed using immunocytochemistry and assessed quantitatively. The mean optical density is measured to indicate protein levels. (mean ± SD, * P < 0.05 , scale bars: 10 μm). [file 13578_2021_682_MOESM5_ESM.pdf]

**Fig. S6: Snail is highly expressed in TNBC and inversely correlated with prognosis.**

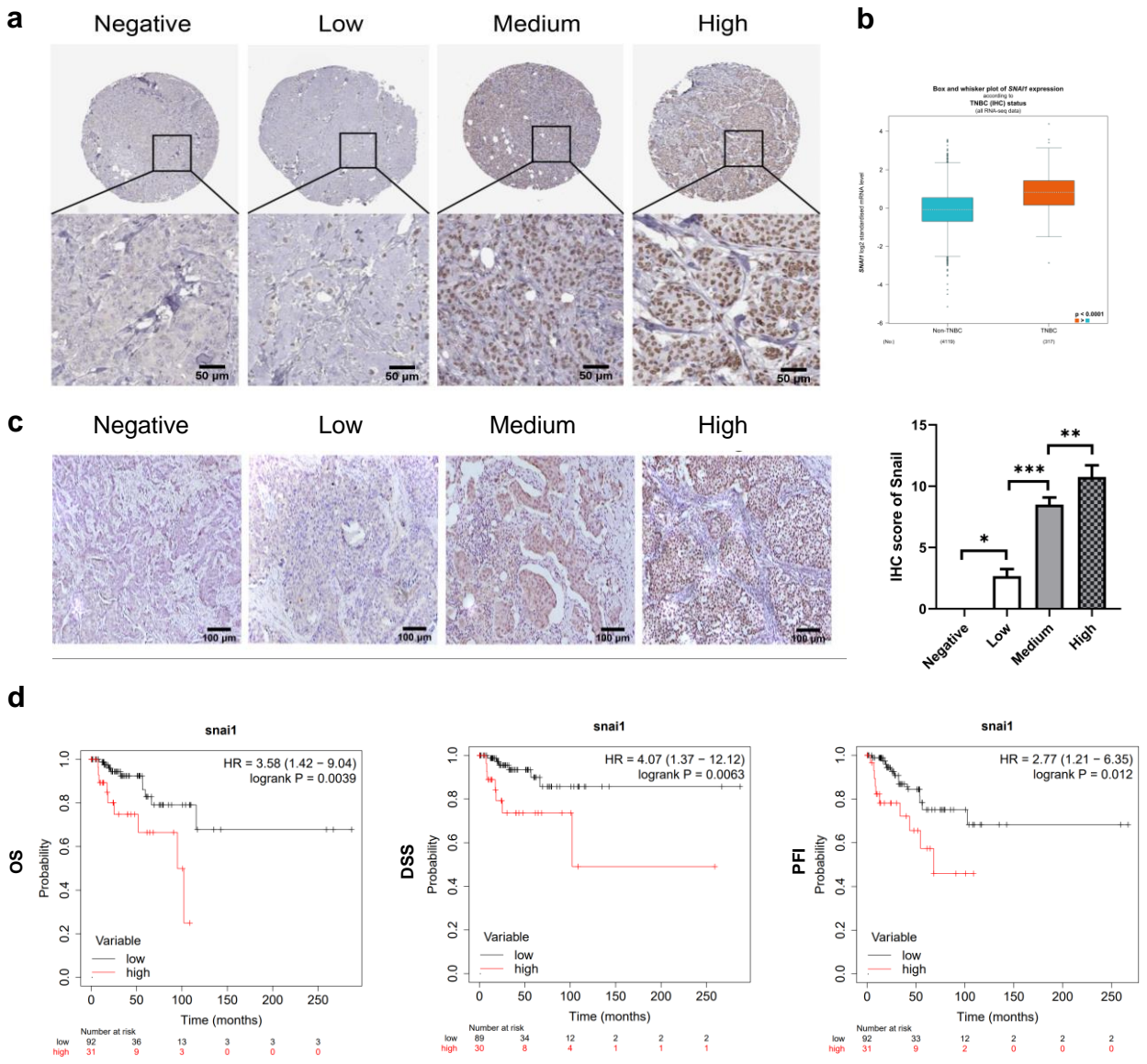

Supplement: Supplementary file 6 — Additional file 6: Figure S6. Snail is highly expressed in TNBC and correlates with cancer relapse. a Representative immunohistochemistry images show Snail protein expression in breast cancer tissues (Human Protein Atlas, scale bars: 50 μm). b Expression of Snail in 13 TNBC tissue samples is detected using immunocytochemistry. Representative images of Snail staining in breast cancer tissue; magnification ×10; scale bars: 100 μm. c Relationship between Snail mRNA expression and different subclasses of breast cancer patients (bc-GenExMiner 4.5) (* P < 0.05, ** P < 0.01, and *** P < 0.001). d The prognostic values of the mRNA expression of Snail in TNBC patients are analyzed (Kaplan-Meier plotter). The top quartile was used as the cutoff value. [file 13578_2021_682_MOESM6_ESM.pdf]
