# Supplementary material for: Huaier polysaccharides suppress triple-negative breast cancer metastasis and epithelial-mesenchymal transition by inducing autophagic degradation of Snail
Source: Cell Biosci. 2021 Sep 4;11:170. doi: 10.1186/s13578-021-00682-6 (PMC8417980; doi:10.1186/s13578-021-00682-6)

Fig. S2: Expression of autophagy-related markers is positively correlated with E-cadherin expression.

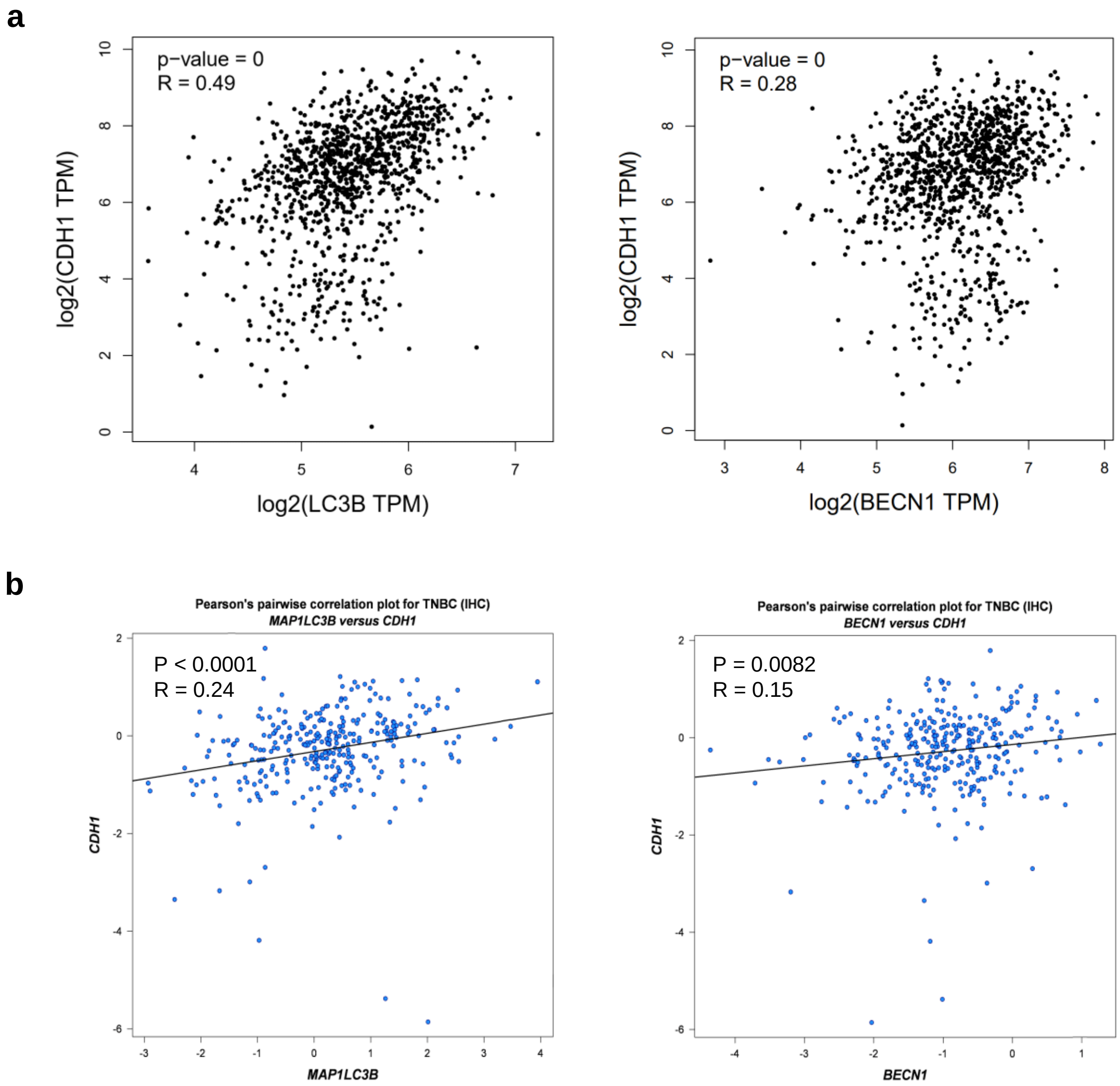

Supplement: Supplementary file 2 — Additional file 2: Figure S2. mRNA expression of LC3B and Beclin-1 is positively correlated with that of E-cadherin in breast cancer. Using GEPIA 2.0 and bc-GenExMiner 4.5 to analyze TCGA databases, we found that the mRNA expression of the autophagy-related markers LC3B and Beclin-1 was positively correlated with E-cadherin expression in all breast cancer (a) and TNBC patients (b). [file 13578_2021_682_MOESM2_ESM.pdf]
